# Exergy Analysis of Fluidized Desiccant Cooling System

**DOI:** 10.3390/e21080757

**Published:** 2019-08-02

**Authors:** Zbigniew Rogala, Piotr Kolasiński

**Affiliations:** Department of Thermodynamics, Theory of Machines and Thermal Systems, Wrocław University of Science and Technology, Wybrzeże Wyspianskiego 27, 50-370 Wrocław, Poland

**Keywords:** fluidized bed, desiccant cooling, adsorption, exergy destruction, exergy efficiency

## Abstract

One of the main challenges in the design and implementation of fluidized desiccant cooling (FDC) systems is increasing their low COP (coefficient of performance). Exergy analysis is one of the tools especially suitable for improvement and optimization of FDC systems. The improvement of performance is impossible as long as the main sources of exergy destruction are not identified and evaluated. In this paper, the exergy analysis was applied in order to identify these components and processes of the FDC system that are mainly responsible for exergy destruction. Moreover, the exergy efficiency of a simple fluidized desiccant cooler was determined. The results showed that fluidized beds and regenerative heat exchanger were the main exergy destruction sources with a 32% and 18% share of total exergy destruction, respectively. On the other hand, the direct evaporative cooler and air cooler placed after the desorbing fluidized bed were characterized by the lowest exergy efficiencies. This work contributes to better understanding of FDC operation principles and improvement of the performance of FDC technology.

## 1. Introduction

Increasing global energy consumption and strict policy related to energy efficiency led to intensified R&D works on energy systems powered by renewable or waste energy sources including solid [1,2,3,4,5] and liquid [6,7,8] desiccant cooling systems. According to [9], one can divide solid desiccant cooling systems into honeycomb desiccant wheel systems [10,11], packed bed systems [2], and fluidized bed systems [1,3]. The application of fluidized beds in solid desiccant cooling systems results in decreased bed pressure drop [3] and improved heat and mass transfer performance [12] in comparison with the packed bed systems. Moreover, fluidized bed systems are less expensive than desiccant wheel systems [9]. Therefore, the development of the fluidized desiccant cooling technology is nowadays of great interest. The modeling methods of adsorption/desorption under fluidization, useful for fluidized desiccant cooling systems’ design, were provided by [3,5,13,14]. Chen [3], Horibe [4], and Rogala [14] carried out experimental analyses of air dehumidification in fluidized beds. Rogala [1] carried out a theoretical analysis of the effect of the operating parameters of the fluidized beds on the performance of the system and provided recommendations on efficient running of fluidized desiccant cooling (FDC). Finally, Chen [2] and Chiang [9] presented prototype self-circulating fluidized beds, which reduce electric power consumption. Despite the above-mentioned scientific works, fluidized desiccant cooling is still at the early stage of development. In the authors’ opinion, the main challenge is to increase the low COP of fluidized desiccant cooling systems, which according to the theoretical investigation presented in [1], unlikely exceeds 0.6. However, cooling systems based on adsorption are according to [15] limited to a COP of 0.9, which results from the physics of adsorption. The difference of the performance between actual [1] and ideal [15] systems comes from the irreversibilities of the processes in FDC. Identification and mitigation of these irreversibilities would result in a significant increase of COP. Therefore, in the authors’ opinion, such analysis should be carried out. A tool reported as meaningful and reliable in such considerations is exergy analysis [6,8,11,16,17]. This paper presents the results of the exergy analysis of a fluidized desiccant cooling system. The main aim of the analysis was to point out the components mostly responsible for the exergy destruction in FDC. Moreover, the exergy losses were also classified with respect to their origin: exergy losses accompanying heat transfer and exergy losses connected with the flow and related to humidity changes.

## 2. Fluidized Desiccant Cooling System

**Figure 1:**
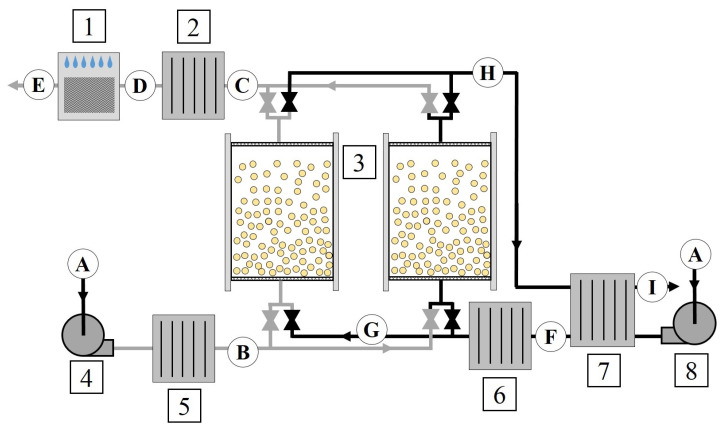
Scheme of the analyzed fluidized desiccant cooling system: 1, direct evaporative cooler (DEC); 2, air cooler ($AC_{2}$); 3, fluidized beds (FB); 4, electric fan AF; 5, air cooler ($AC_{1}$); 6, air heater (AH); 7, regenerative heat exchanger (RHX); 8, electric fan (DF). Letters A–I correspond to the air characteristic point of the process (see, Figure 2).

**Figure 2:**
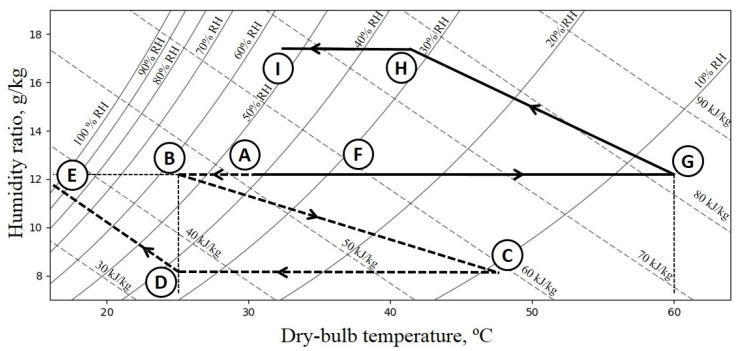
Psychrometric processes taking place in fluidized desiccant cooling (FDC).

The principles of operation of FDC were comprehensively described in [1]. The scheme of the analyzed fluidized desiccant cooler is presented in Figure 1. Additionally, all the processes taking place in FDC are presented in Figure 2. The working fluid of the system is a mixture of dry air and water vapor; therefore, all the processes are psychrometric. The aim of the system is to cool down the air by initial desiccation (using fluidized beds) and final rehumidification, which makes air cooler due to water evaporation. The system consists of two fluidized beds filled with a desiccant (in this case silica gel), which are periodically supplied from either a hot air or cold air section. In cold the air section, the fan (4) blows the air through the air cooler (A–B). The air coolers (2) and (5) are supplied with cooling water from wet cooling towers. Therefore, it is possible to obtain water with a temperature ranging between ambient and wet-bulb temperature (which for the given ambient conditions is about 22 ${}^{\circ }$C). Then, the air enters the fluidized bed (3). Due to adsorption, the air is dehumidified and warmed up (B–C). The dehumidified air is cooled again in the air cooler close to ambient temperature (C–D) and, at last, it is humidified in a direct evaporative cooler (DEC) (Processes D–E), which results in a temperature drop. Simultaneously, in the second fluidized bed, the desiccant is regenerated. In the hot air section, the fan (8) blows the air through the recovery heat exchanger (A–F) and the air heater (F–G). The air is heated in the air heater thanks to the heat supplied by warm water, the temperature of which is about 65 ${}^{\circ }$C (therefore, e.g., industrial low-grade waste heat sources can be applied as a heat source). Then, the hot air enters the fluidized bed, where due to its high temperature, desorption of water from the desiccant takes place (G–H). At last, the air flowing out from the desorbing fluidized bed flows again through the recovery heat exchanger (H–I).

## 3. Exergy Analysis

Exergy analysis is based on exergy balance, which can be described by the following equation, resulting from the second law of thermodynamics [18]:

(1) $$\sum_{in}{\dot{Ex}}_{\dot{Q}}+\sum_{in}\dot{m}\left(ex\right)-\sum_{out}{\dot{Ex}}_{\dot{Q}}-\sum_{out}\dot{m}\left(ex\right)-{\dot{Ex}}_{dest}=0$$

In Equation (1), kinetic and potential energies and work are assumed to be negligible. The exergy balance equation takes into account input and output exergies as heat or mass flows. Due to the irreversibility of thermodynamic processes, the input and output exergies are not equal, which is expressed by exergy destruction ${\dot{Ex}}_{dest}$. According to [18], the general definition of specific exergy is expressed as:

(2) $$ex=h-h_{0}-T_{0}\left(s-s_{0}\right)$$

However, for the psychrometric processes, the exergy is expressed as [18]:

(3) $$ex=\left(c_{p,a}+Xc_{p,v}\right)T_{0}\left(\frac{T}{T_{0}}-1-ln\left(\frac{T}{T_{0}}\right)\right)+\left(1+\bar{X}\right)R_{a}T_{0}ln\left(\frac{p}{p_{0}}\right)+R_{a}T_{0}\left(\left(1+\bar{X}\right)ln\left(\frac{1+\bar{X_{0}}}{1+\bar{X}}\right)+\bar{X}ln\left(\frac{\bar{X}}{\bar{X_{0}}}\right)\right)$$

The parameters assigned with subscript “0” are related to the dead state, which is a reference state for exergy calculation. The dead state conditions are listed in Table 1. The effectiveness of particular processes and components of the system is evaluated by means of exergy efficiency $\eta_{ex}$, which is expressed as [18]:

(4) $$\eta_{ex}=1-\frac{{\dot{Ex}}_{dest}}{\sum_{in}{\dot{Ex}}_{\dot{Q}}+\sum_{in}\dot{m}\left(ex\right)}$$

In order to determine the exergy efficiency, all of the exergy inputs and outputs have to be identified, and the exergy balance needs to be solved. The total exergy of humid air (see, Equation (3)) consists of three components, i.e., exergy related to the temperature, pressure, and humidity of air, respectively. All of the processes that are taking place in the fluidized desiccant cooling system are described in the following subsections by means of the exergy balance equation in order to evaluate the irreversibility involved in these processes.

### 3.1. Electric Fans

**Figure 3:**
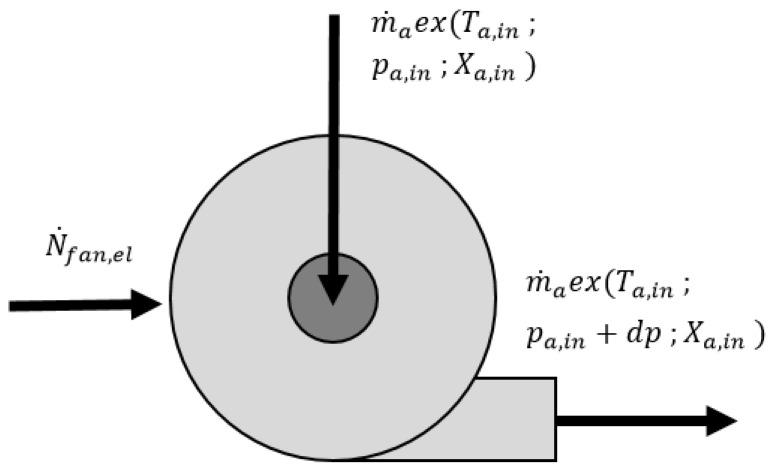
Exergy and flow scheme of the electric fan.

The electric fans (see, Figure 3) provide the required pressure increase, which is equal to the total pressure drop of the system. The exergy destruction of the electric fan was estimated based on the following assumptions:

1. The electric fan has an isentropic efficiency of 70% (such efficiency was justified by [19]).
2. The increase of the exergy of the air compressed in the electric fan is only due to the pressure rise (the temperature-related exergy is neglected).

The exergy balance equation of the electric fan is as follows:

(5) $${\dot{N}}_{fan,el}+{\dot{m}}_{a}ex\left(T_{a,in},p_{a,in},X_{a,in}\right)-{\dot{m}}_{a}ex\left(T_{a,out},p_{a,out},X_{a,out}\right)-Ex_{dest,fan}=0$$

As exergy is the potential to perform work, the mechanical power delivered to the fan ${\dot{N}}_{fan,el}$ is pure exergy flow.

### 3.2. Air Heater/Cooler

**Figure 4:**
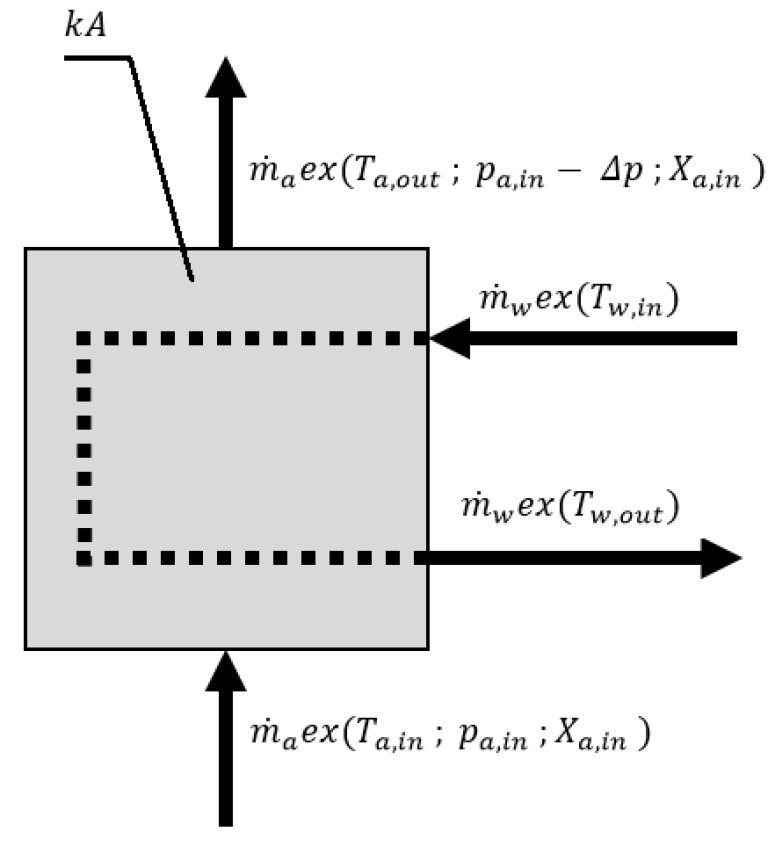
Exergy and flow scheme of the air heater/cooler.

The air heater/cooler (see, Figure 4) is used both during adsorption and desorption modes. The air passing through this heat exchanger is either heated or cooled by water flow. In order to estimate the exergy destruction in the air heater/cooler, the following assumptions were applied:

1. The outer wall of the heat exchanger is adiabatic,
2. The heat capacity of the heat exchanger is negligible,
3. The water pressure drop in the heat exchanger is negligible,
4. Condensation of water vapor on the heat exchange surfaces does not occur,
5. In the case of the air cooler, the exergy of water at the outlet is dissipated.

The exergy balance equation for the air heater/air cooler is the following:

(6) $${\dot{m}}_{a}ex\left(T_{a,in},p_{a,in},X_{a,in}\right)+{\dot{m}}_{w}ex\left(T_{w},in\right)-{\dot{m}}_{a}ex\left(T_{a,out},p_{a,out},X_{a,out}\right)-{\dot{m}}_{w}ex\left(T_{w},out\right)-Ex_{dest,AC/AH}=0$$

The exergy destruction occurs due to the air pressure drop and finite temperature difference during heat transfer. The heat transfer equation in the air cooler/heater can be described as:

(7) $$\dot{Q}=F\cdot k\cdot A\cdot LMTD$$

where LMTD is expressed as:

(8) $$LMTD=\frac{\left(T_{w,out}-T_{a,in}\right)-\left(T_{w,in}\;-T_{a,out}\right)}{ln\left(\frac{T_{w,out}\;-\;T_{a,in}}{T_{w,in}\;-\;T_{a,out}}\right)}$$

The heat balance equations of water and air streams are as follows:

(9) $$\dot{Q}={\dot{m}}_{w}c_{w}\left(T_{w,in}-T_{w,out}\right)$$

(10) $$\dot{Q}={\dot{m}}_{a}c_{a}\left(T_{a,out}-T_{a,in}\right)$$

By introducing Equations (9) and (10) into Equation (8) and then combining the achieved result with Equation (7), one can derive the expression describing the temperature of the air at the outlet of the air heater/cooler:

(11) $$T_{a,out}=\frac{T_{w,in}\left(E-1\right)-T_{a,in}\left(R_{c}-1\right)}{E-R_{c}}$$

where $R_{c}$ is a heat capacity ratio:

(12) $$R_{c}=\frac{{\dot{m}}_{a}c_{a}}{{\dot{m}}_{w}c_{w}}$$

and *E* is given by the following expression:

(13) $$E=exp(NTU_{a}\left(1-R_{c}\right))$$

where $NTU_{a}$ describes the geometry of the heat exchanger and is expressed as:

(14) $$NTU_{a}=\frac{kAF}{{\dot{m}}_{a}c_{a}}$$

The cross-flow of the air and the water occurs in the heat exchanger; thus, the correction factor *F* must be applied. According to [20], this correction factor can be obtained from the following relation:

(15) $$F=\frac{ln\left(\frac{1-R_{c}P}{1-P}\right)}{NTU_{w}\left(\frac{1}{R_{c}}-1\right)}$$

where *P* is a temperature ratio expressed as:

(16) $$P=\frac{T_{a,out}-T_{a,in}}{T_{w,in}-T_{a,in}}$$

and $NTU_{w}$ is expressed as:

(17) $$NTU_{w}=\frac{kAF}{{\dot{m}}_{w}c_{w}}$$

At last, the temperature of the water at the outlet of the heat exchanger can be calculated based on the following energy balance:

(18) $$T_{w,out}=T_{w,in}-R_{c}\left(T_{a,out}-T_{a_{i}n}\right)$$

Equations (11)–(18) need to be solved iteratively. Assuming that the velocity of air inside the heat exchanger is equal to 3 ${\mathrm{ms}}^{-1}$ and the overall heat transfer coefficient is equal to 50 ${\mathrm{Wm}}^{-2}K^{-1}$ [21], the air pressure drop Δp can be calculated as:

(19) $$\Delta p_{AC/AH}=\frac{32\cdot U^{2}\rho_{a}^{2}A\nu }{dm_{a}}$$

Due to the applied assumptions, the presented methodology can be applied either in the case of the constant temperature of the air at the inlet to the heat exchanger (see Component (5) in Figure 1) or in the case of transient inlet conditions (see Components (2) and (6) in Figure 1).

### 3.3. Regenerative Heat Exchanger

**Figure 5:**
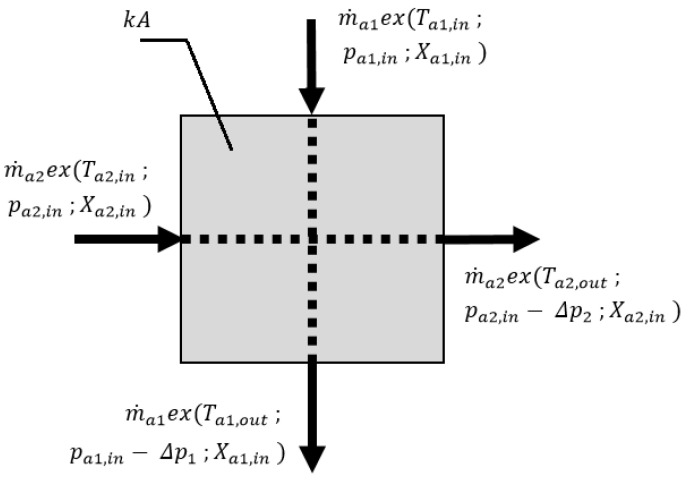
The exergy and flow scheme of the regenerative heat exchanger (RHX).

After desorption, the air passes through the regenerative heat exchanger (RHX) before it is released to the surroundings. Due to transient inlet conditions, it is recommended to apply a crossflow air-to-air heat exchanger rather than a heat wheel (which requires steady inlet conditions) as RHX. The exergy and flow scheme of RHX is presented in Figure 5.

The air passes through the RHX (see Component (7) in Figure 1) before it enters the air heater (see Component (6) in Figure 1). The RHX is fed from the outlet of the desorbing fluidized bed (see Component (3) in Figure 1); therefore, it operates in transient conditions. In order to estimate the exergy destruction in the RHX, the following assumptions were applied:

1. The outer wall of the heat exchanger is adiabatic,
2. The heat capacity of the heat exchanger is negligible,
3. Condensation of water vapor on the heat exchange surfaces does not occur.

The exergy balance equation for RHX, due to similar air mass flows and no change in specific humidity ratio, has the following form:

(20) $$ex\left(T_{a1,in},p_{a1,in},X_{a1,in}\right)+ex\left(T_{a2,in},p_{a2,in},X_{a2,in}\right)-ex\left(T_{a1,out},p_{a1,in}-\Delta p_{1},X_{a1,in}\right)-ex\left(T_{a2,out},p_{a2,in}-\Delta p_{2},X_{a2,in}\right)-Ex_{dest,RHX}=0$$

The temperature of the air at the outlet of the RHX can be calculated from the energy balance equation, which leads to the following expression:

(21) $$T_{a1,out}=\frac{{\dot{m}}_{a}c_{a}T_{a1,in}+kAFT_{a2,in}}{kAF+{\dot{m}}_{a}c_{a}}$$

Due to the cross-flow of the air streams in the RHX, a correction factor *F* needs to be applied. According to [21], this correction factor depends on the temperature ratio *P* (see Equation (16)) and the heat capacity ratio $R_{c}$. In the analyzed case, $R_{c}$ was close to one due to the same mass flows of dry air at both sides of the heat exchanger. Therefore, the correction factor can be expressed as [21]:

(22) $$F=-2.6224\times P^{4}+2.2429\times P^{3}-0.9911\times P^{2}+0.073\times P+1.0003$$

Equations (21) and (22) need to be solved iteratively. Assuming that the air velocity in both channels is equal to 5 ${\mathrm{ms}}^{-1}$ and the overall heat transfer coefficient is equal to 25 ${\mathrm{Wm}}^{-2}K^{-1}$ (which is consistent with experimental and theoretical studies [22,23,24]), the air pressure drop generated in the RHX is expressed as:

(23) $$\Delta p_{RHX}=12.5\frac{U^{2}\rho_{a}^{2}A\nu }{\left(w+l\right)\cdot m_{a}}$$

where *w* and *l* are the dimensions of a single RHX channel.

### 3.4. Fluidized Beds

**Figure 6:**
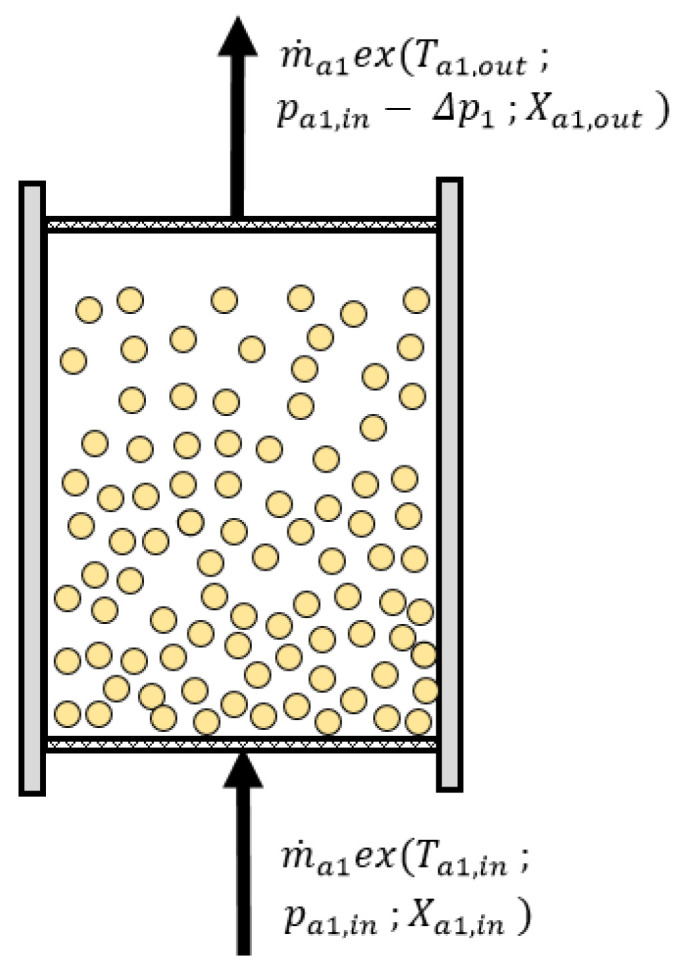
Exergy and flow scheme of the fluidized bed.

The fluidized beds are the main components of the fluidized desiccant cooling system (see component (3) in Figure 1). The exergy and flow scheme of the fluidized bed is presented in Figure 6.

The fluidized bed is supplied with pretreated (cooled or heated) air in order to initiate adsorption or desorption. The exergy balance includes only exergy streams of the air at the inlet and at the outlet of the fluidized bed. However, loose desiccant placed in the bed undergoes periodic changes of temperature and water uptake. It accumulates sensible (by temperature growth of the desiccant) and latent heat (by water uptake growth of the desiccant), and therefore, it can accumulate exergy. During desorption, the desiccant gets rid of moisture, and it is heated up. After adsorption is started, latent and sensible heats are released back to the adjacent air stream. Taking into account the periodicity of sorption processes, adsorption and desorption are analyzed simultaneously. Otherwise, exergy gain would be observed during adsorption (due to release of the desiccants’ exergy), and excessive exergy destruction would be observed during desorption (due to the accumulation of the exergy in the desiccant). The change of the air stream properties at the outlet of the fluidized bed and the pressure drop of the air were evaluated according to the methodology presented in [14]. The exergy balance accounted together for the adsorbing and desorbing fluidized bed is as follows:

(24) $${\dot{m}}_{a1}ex\left(T_{a1,in},p_{a1,in},X_{a1,in}\right)+{\dot{m}}_{a2}ex\left(T_{a2,in},p_{a2,in},X_{a2,in}\right)-{\dot{m}}_{a1}ex\left(T_{a1,out},p_{a1,in}-\Delta p_{a1},X_{a1,out}\right)-{\dot{m}}_{a2}ex\left(T_{a2,out},p_{a2,in}-\Delta p_{a2},X_{a2,out}\right)-Ex_{dest,FB}=0$$

### 3.5. Direct Evaporative Cooler

**Figure 7:**
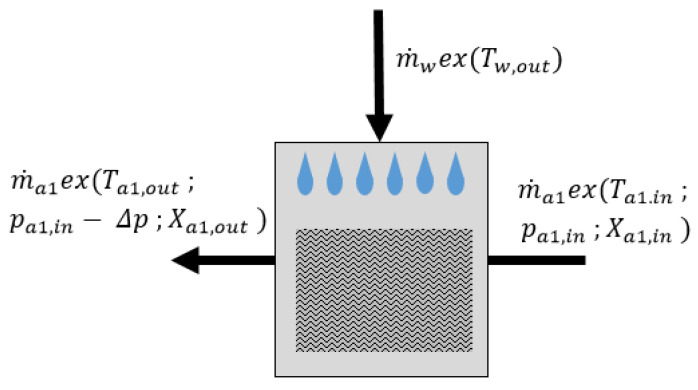
Exergy and flow scheme of the direct evaporative cooler (DEC).

The exergy and flow scheme of a direct evaporative cooler is presented in Figure 7. Initially, the air flowing through DEC cools down (theoretically reaching the wet-bulb temperature), due to direct contact with the evaporating water. According to [25], the wet-bulb temperature is not actually reached. In order to evaluate the exergy destruction in DEC, the following assumptions were applied:

1. The outer walls of the DEC are adiabatic,
2. The heat capacity of the DEC is negligible,
3. The water stream supplied to the DEC undergoes complete evaporation,
4. DEC generates a constant pressure drop of 25 Pa [26],
5. The heat of evaporation is exchanged at the temperature level of the outlet air.

Based on the above-mentioned assumptions, exergy destruction can be estimated as:

(25) $${\dot{Ex}}_{dest}={\dot{m}}_{a}\cdot ex_{a,in}+{\dot{m}}_{w}\cdot \left(ex_{w,in}+H_{ev}\cdot \left(1-\frac{T_{0}}{T_{a,out}}\right)\right)-{\dot{m}}_{a}ex_{a,out}$$

Besides the exergy streams presented in Figure 7, the exergy of evaporation of the sprayed water needs to be taken into account. It was assumed that the total heat of evaporation is exchanged at the outlet air temperature level $T_{a,out}$.

## 4. Results and Discussion

The presented methodology was applied to FDC components in order to identify the exergy destruction sources and assess the limits of the exergy efficiency of the FDC components. Firstly, the exergy efficiency of all the components (beside fluidized beds) was investigated separately to estimate their influence on the total exergy destruction. Then, the exergy destruction of the complete FDC system was analyzed.

### 4.1. Air Cooler

**Figure 8:**
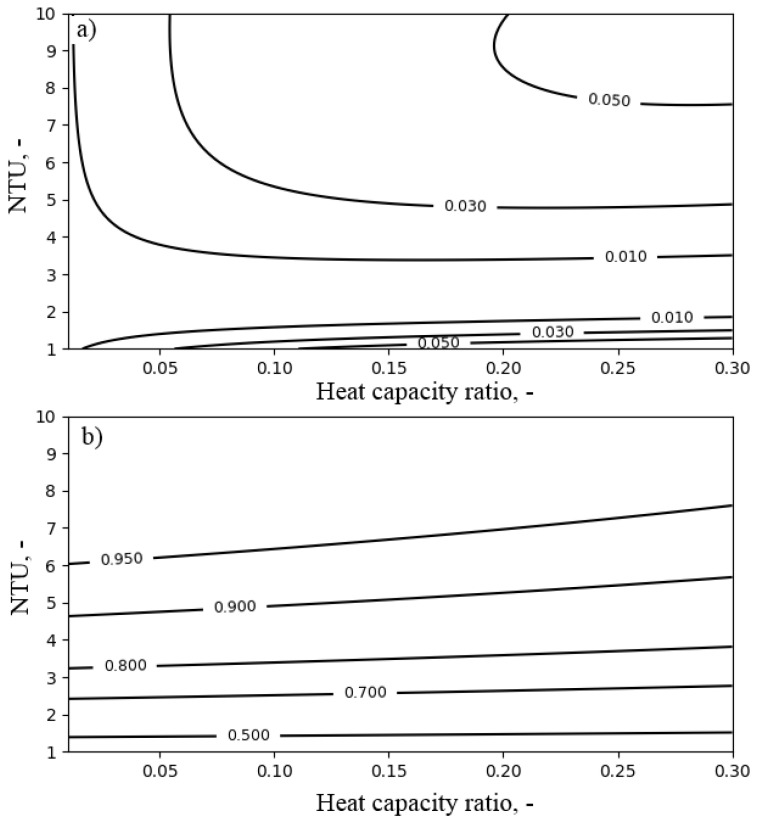
The influence of $NTU_{a}$ and heat capacity ratio $R_{c}$ on the performance of the air cooler for the inlet air temperature $T_{a,in}$ = 40 ${}^{\circ }C$: (**a**) exergy efficiency; (**b**) temperature ratio.

The performance of the air cooler depended on the heat capacity ratio $R_{c}$ (see Equation (12)), $NTU_{a}$ (see Equation (14)) and the inlet air temperature $T_{a,in}$. Therefore, the influence of these parameters on the exergy efficiency of the air cooler was investigated. The influence of the heat capacity ratio $R_{c}$ and $NTU_{a}$ on the exergy efficiency and the temperature ratio of the air cooler is presented in Figure 8. The temperature of the air at the inlet to the air cooler was set to 40 ${}^{\circ }$C.

**Figure 9:**
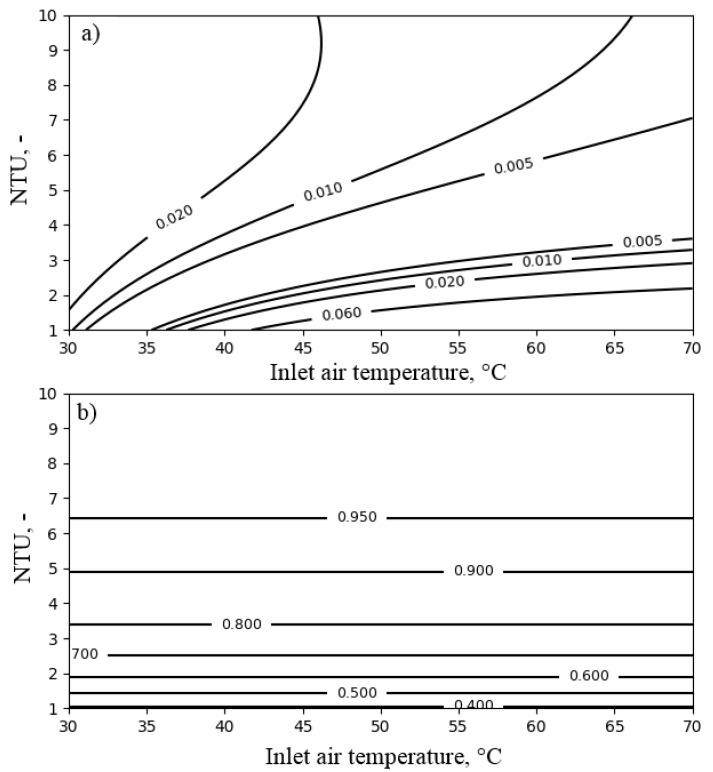
The influence of $NTU_{a}$ and the inlet air temperature $T_{a,in}$ on the performance of the air cooler for heat capacity ratio $R_{c}$ = 0.1: (**a**) exergy efficiency; (**b**) temperature ratio.

As can be seen in Figure 8, the exergy efficiency of the air cooler did not exceed the value of 0.06. This resulted from the dissipation of exergy accumulated in the outlet water. By increasing the $NTU_{a}$ value above six, the temperature ratio exceeding the value of 0.9 and exergy efficiency of 0.03 could be obtained. Increasing the heat capacity ratio improved the exergy efficiency up to 0.05. Figure 9 presents the influence of $NTU_{a}$ and the temperature of the air at the inlet to the air cooler $T_{a,in}$ on FDC performance. As can be observed from Figure 9, for $NTU_{a}$ values higher than four, the lower the inlet air temperature was, the higher the exergy efficiency obtained. Nevertheless, the increase of efficiency was slight. On the other hand, the temperature ratio was irrespective of the temperature of the air at the inlet to the air cooler, and its values remained above 0.9 for $NTU_{a}$ values exceeding six.

**Figure 10:**
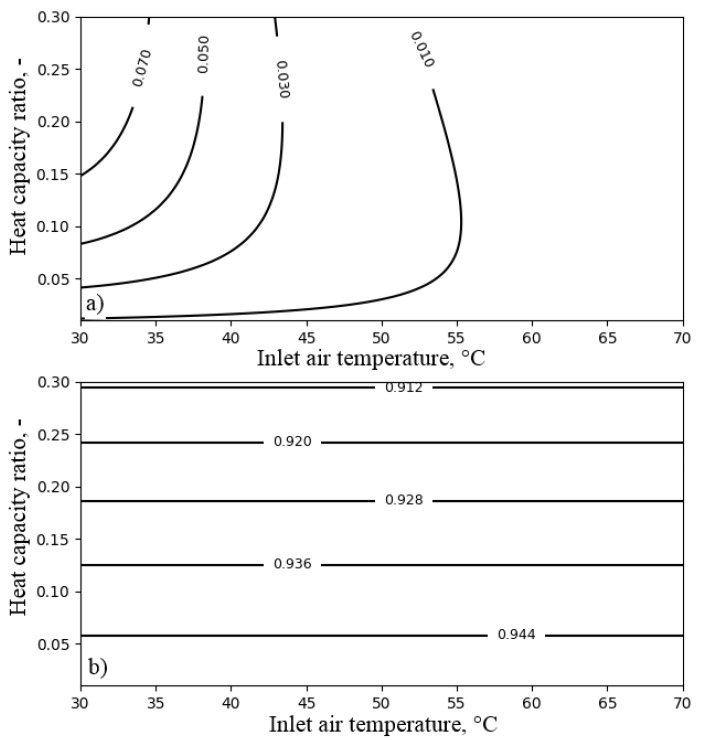
The influence of the heat capacity ratio $R_{c}$ and the inlet air temperature $T_{a,in}$ on the performance of the air cooler for $NTU_{a}$ = 6: (**a**) exergy efficiency; (**b**) temperature ratio.

Figure 10 presents the influence of the heat capacity ratio and the temperature of the air at the inlet to the air cooler on its performance. Analysis was carried out for an $NTU_{a}$ value of xis. As can be seen in Figure 10, the decrease of the temperature of the air at the inlet to the air cooler limited the exergy loss. Increasing the heat capacity ratio resulted in increased exergy efficiency, but on the other hand, a decreased temperature ratio.

Due to the dissipation of the exergy of the outlet water, the air cooler was very inefficient from the exergy point of view, and it reached an exergy efficiency of up to 0.07. In order to minimize exergy loss, the exergy of the outlet water should be recovered. Due to the low and transient outlet water temperature, such a recovery may be difficult.

### 4.2. Air Heater

**Figure 11:**
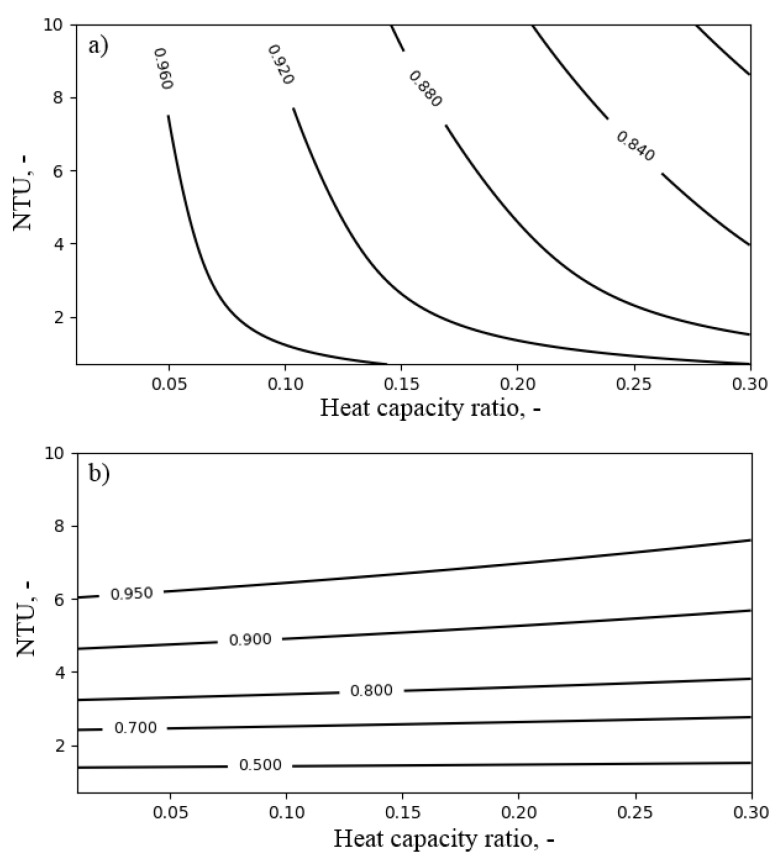
The influence of $NTU_{a}$ and the heat capacity ratio $R_{c}$ for the inlet air temperature $T_{a,in}$ = 40 ${}^{\circ }C$ on the performance of the air heater: (**a**) exergy efficiency; (**b**) temperature ratio.

As was already mentioned, the main difference between air coolers’ and air heaters’ operation is that, in the case of air heaters, the exergy of outlet water is not dissipated. Figure 11 presents the influence of $NTU_{a}$ and the heat capacity ratio $R_{c}$ on the exergy efficiency and the temperature ratio of the air heater. The exergy efficiency increased with decreasing $R_{c}$. Nevertheless, its value remained above 0.8 for the considered range of operating conditions. Increasing $NTU_{a}$ positively influenced the exergy efficiency as long as the benefit from the reduction of the temperature-related exergy destruction was higher than the exergy destruction related to the pressure drop (which increased with increasing $NTU_{a}$). On the other hand, the temperature ratio depended mainly on $NTU_{a}$. Its value was above 0.9 for $NTU_{a}$ larger than six.

**Figure 12:**
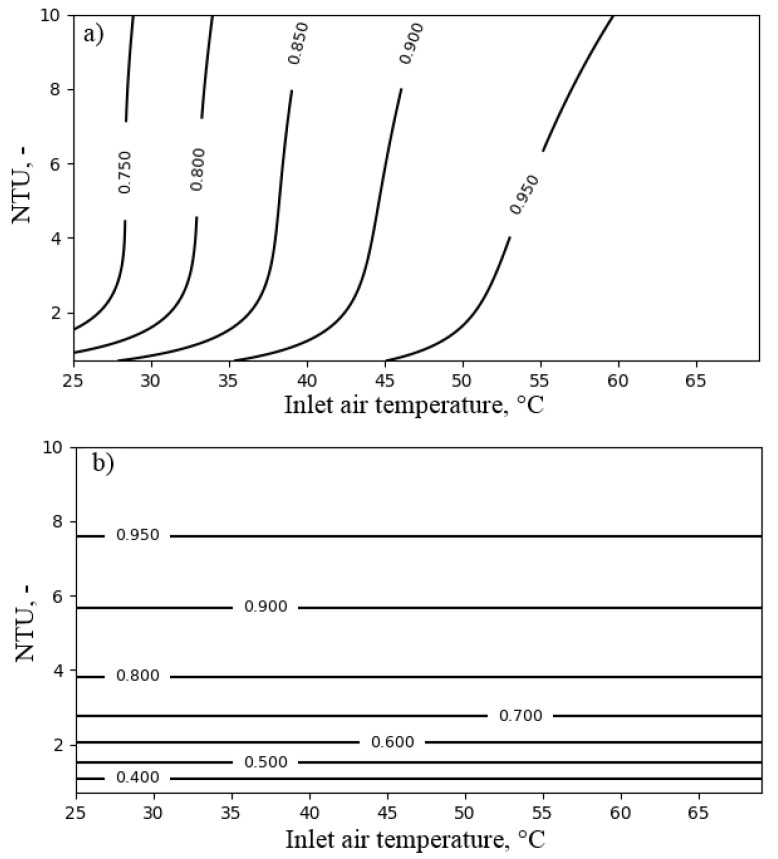
The influence of $NTU_{a}$ and the inlet air temperature $T_{a,in}$ for heat capacity ratio $R_{c}$ = 0.3 on the performance of the air heater: (**a**) exergy efficiency; (**b**) temperature ratio.

Figure 12 presents the influence of $NTU_{a}$ and the inlet air temperature $T_{a,in}$ on the performance of the air heater. The exergy efficiency varied from 0.75 to over 0.95 for the range from 25 ${}^{\circ }C$–70 ${}^{\circ }C$. For the inlet air temperature lower than 45 ${}^{\circ }C$, the influence of $NTU_{a}$ on exergy efficiency was slight. On the other hand, it strongly influenced the temperature ratio.

**Figure 13:**
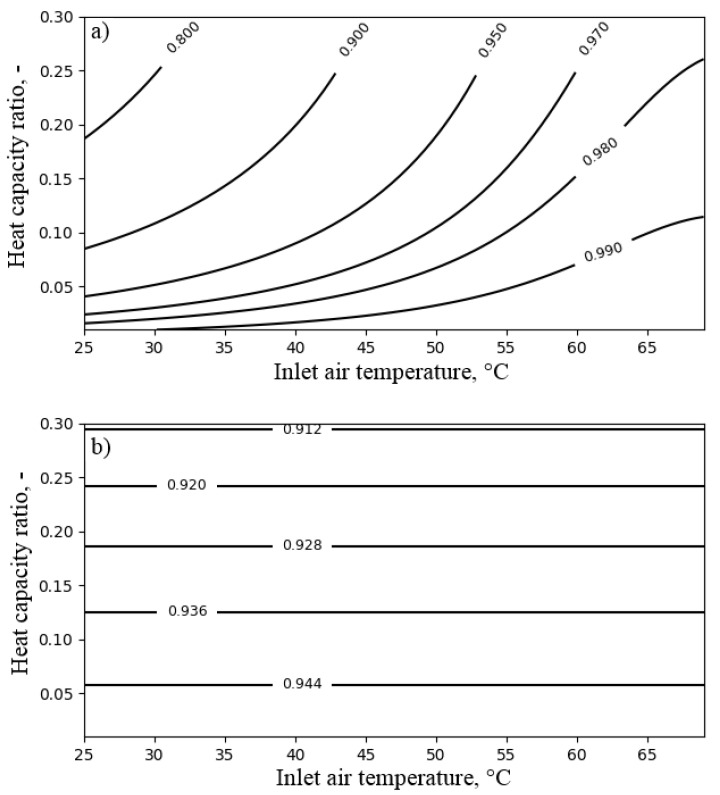
The influence of the heat capacity ratio $R_{c}$ and the inlet air temperature $T_{a,in}$ for $NTU_{a}$ = 6 on the performance of the air heater: (**a**) exergy efficiency; (**b**) temperature ratio.

At last, the performance of the air heater with respect to the heat capacity ratio $R_{c}$ and the inlet air temperature is presented in Figure 13. This confirms the high exergy efficiency and the temperature ratio of the air heater in the considered range of operating conditions.

The air heating process was found to be efficient from the exergy point of view, reaching an exergy efficiency of over 0.7 and a temperature ratio of over 0.9. The important parameter influencing the exergy efficiency of the air heater was the inlet air temperature, which periodically changed due to transient operation of fluidized beds. Moreover, the inlet air temperature depended on the performance of the regenerative heat exchanger.

### 4.3. Regenerative Heat Exchanger

**Figure 14:**
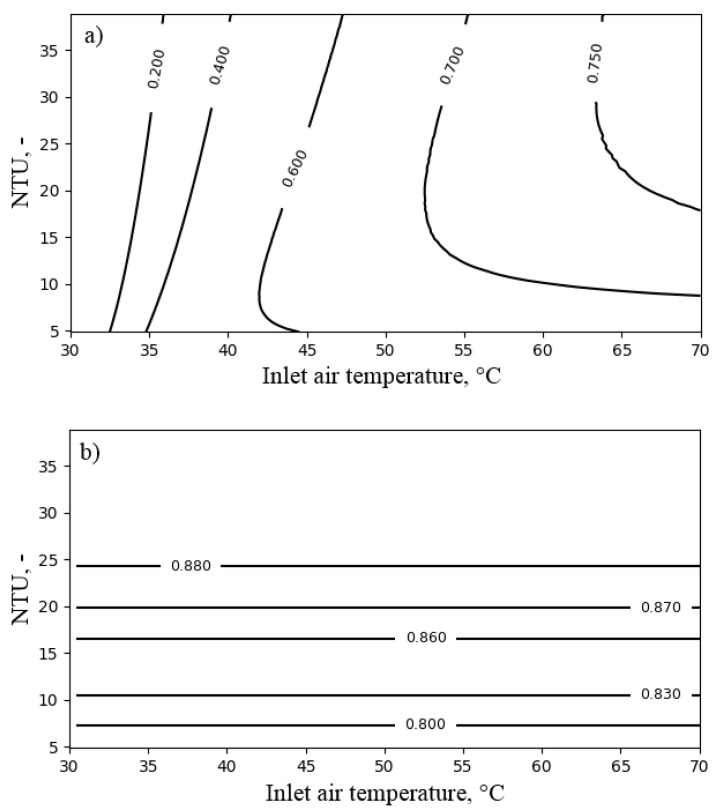
The influence of $NTU_{a}$ and the inlet air temperature $T_{a,in}$ on the performance of the RHX: (**a**) exergy efficiency; (**b**) temperature ratio.

Figure 14 presents the influence of $NTU_{a}$ and the inlet air temperature of the RHX on its performance. The exergy efficiency ranges from 0.2–0.75 and mainly depends on $T_{a,in}$. $NTU_{a}$ seems to have an optimum value, depending on the inlet air temperature. Initially, increasing $NTU_{a}$ allowed regenerating more exergy, approaching the optimum value, but then, exergy efficiency started to decrease. This was due to the excessive pressure drop losses occurring while increasing $NTU_{a}$. On the other hand, the temperature ratio depended only on $NTU_{a}$ and ranged from 0.8–0.9.

### 4.4. Direct Evaporative Cooler

**Figure 15:**
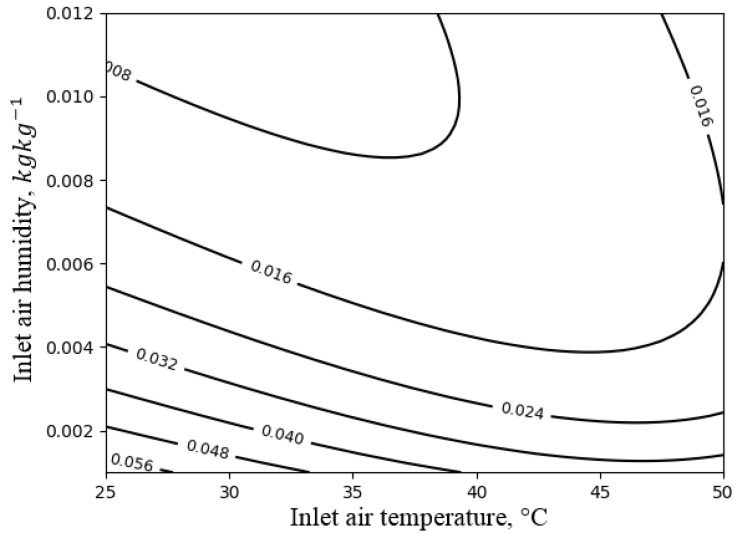
The exergy efficiency of the direct evaporative cooler with respect to the inlet air temperature and humidity.

The influence of the inlet air temperature and humidity on the exergy efficiency of the direct evaporative cooler is presented in Figure 15. The process of evaporative cooling was very inefficient from the exergy point of view. The exergy efficiency did not exceed 0.06. Such poor performance of the DEC was already reported in [26]. One of the possible ways to improve this performance may be the application of an indirect evaporative cooler based on the so-called M-cycle [26,27,28]. As was reported in the literature, the application of the indirect evaporative cooler can improve the exergy efficiency by up to 0.5, and the application of a combined indirect/direct evaporative cooler can increase it up to 0.6 [26], but at the expense of the capacity of the system, which can be reduced even by 40% [27].

### 4.5. Complete System

In the following, the exergy analysis of the complete system is presented. Generally, the FDC system operated in unsteady state as the operation of each of the components of the system influenced the operation of the others. In most of the cases, the parameters of the air at the inlet to the system varied with time. Thus, the exergy efficiency and the exergy destruction were not constant. The parameters of the heat exchangers used in the following analysis are listed in Table 1, while the parameters used for modeling the fluidized beds’ performance (based on [14]) are listed in Table 2.

**Table 1:** Parameters used during the exergy analysis.

Name	Symbol	Unit	Quantity
Dead state humidity	$X_{0}$	${\mathrm{kgkg}}^{-1}$	0.012
Dead state pressure	$p_{0}$	Pa	101300
Dead state temperature	$T_{0}$	${}^{\circ }C$	30
Distance between the AC/AH fins	*d*	m	0.003
Overall heat transfer coefficient of AC/AH	$k_{AC/AH}$	${\mathrm{Wm}}^{-2}K^{-1}$	50
Overall heat transfer coefficient of RHX	$k_{RHX}$	${\mathrm{Wm}}^{-2}K^{-1}$	25
Dimension of the RHX channel	$l_{RHX}$	m	0.01
Dimension of the RHX channel	$w_{RHX}$	m	0.003
Number of heat transfer units of AC/AH	$NTU_{AC/AH}$	−	6
Number of heat transfer units of RHX	$NTU_{RHX}$	−	20
Heat capacity ratio of AC/AH	$R_{c,AC/AH}$	−	0.2
Air velocity in heat exchangers	*U*	${\mathrm{ms}}^{-1}$	3

**Figure 16:**
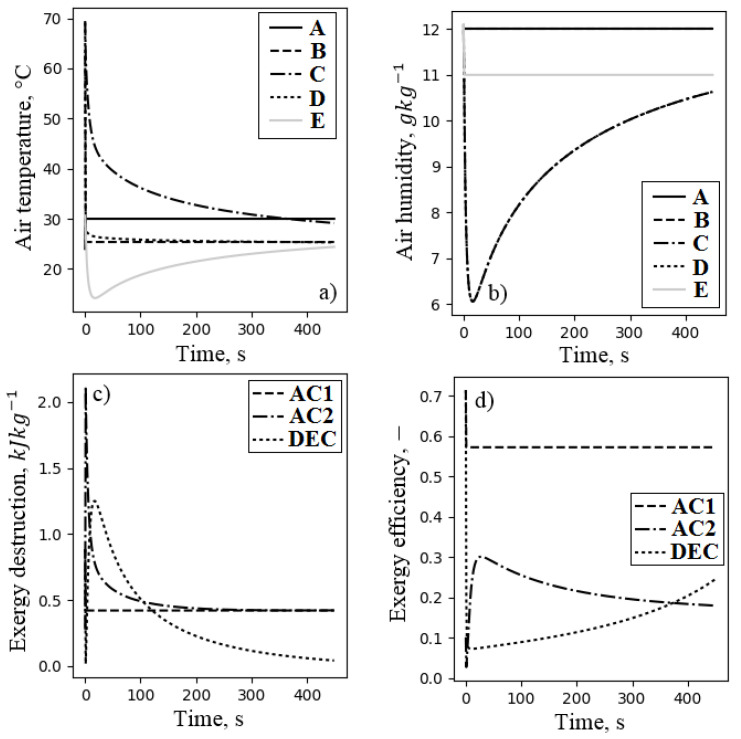
Distribution of the air temperature (**a**), air humidity (**b**), exergy destruction (**c**), and exergy efficiency (**d**) of the FDC during adsorption. Letters corresponds to characteristic points of the processes (see, Figure 2).

**Figure 17:**
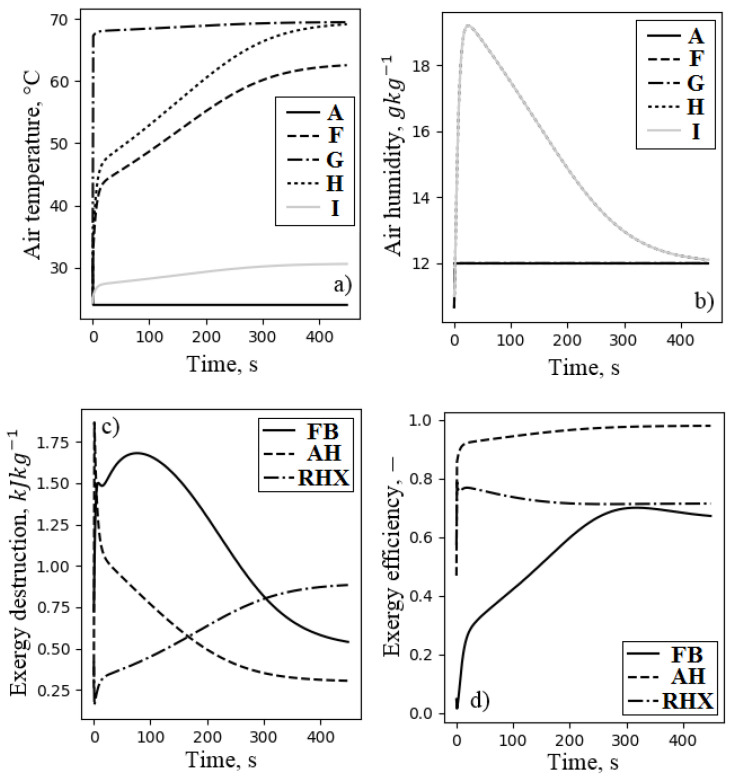
Distribution of air temperature (**a**), air humidity (**b**), exergy destruction (**c**), and exergy efficiency (**d**) of the FDC during desorption. Letters corresponds to air characteristic points (see Figure 1).

**Figure 18:**
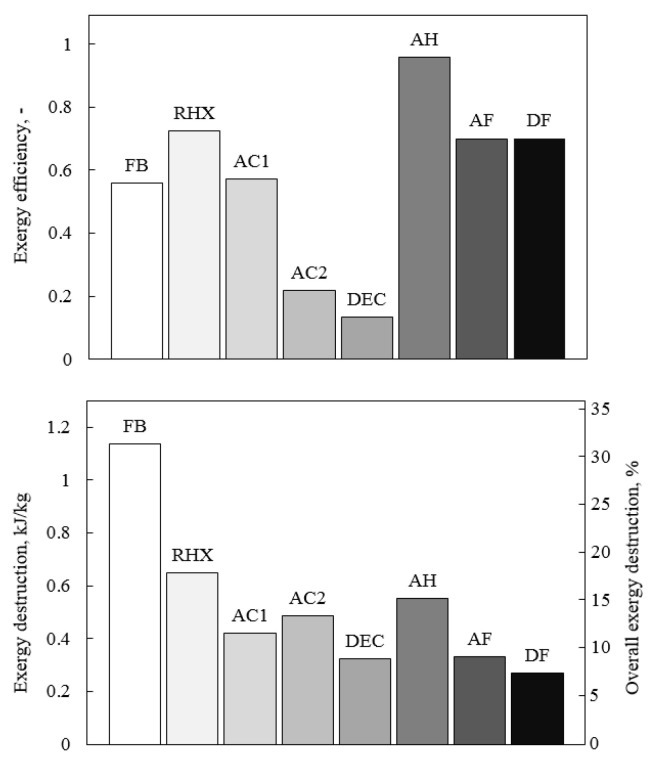
Exergy destructions and exergy efficiencies of particular FDC components: FB, fluidized beds; RHX, regenerative heat exchanger; AC1, first air cooler (see Figure 1(5)); AC2, second air cooler (see Figure 1(2)); DEC, direct evaporative cooler; AH, air heater.

The distribution of the air temperature, the air humidity, the exergy destruction, and the exergy efficiency of all components of the system during adsorption is presented in Figure 16. The highest peak of the exergy destruction was observed during air cooling in $AC_{2}$. At the beginning of adsorption, due to the high temperature of the outlet air from the fluidized bed, exergy destruction was the highest; however, it decreased in time as the outlet air temperature became lower. The air cooler $AC_{2}$ was inefficient with exergy efficiency not exceeding 30%. Another inefficient component was the direct evaporative cooler. The process of evaporation in the DEC increased the effectiveness (lower exergy destruction and higher exergy efficiency) as less water was evaporated. Nevertheless, the exergy efficiency did not exceed 30%. Preliminary air cooler $AC_{1}$ was characterized by constant exergy destruction of less than 0.5 ${\mathrm{kJkg}}^{-1}$ and exergy efficiency of about 60%. This heat exchanger dissipated only the excess of exergy from the ambient environment to the heat sink temperature, which was usually not high. Figure 17 presents the distribution of the temperature and the humidity of the air, as well as the exergy destruction and exergy efficiency of FDC during desorption. This figure reports the performance of both fluidized beds, which are responsible for significant exergy destruction (see Figure 17c). At the beginning of each mode (adsorption or desorption), the exergy efficiency of the fluidized beds was about 5%, and it increased gradually, finally reaching about 60%. Initially, the air heating process was inefficient, but it quickly improved, finally reaching the exergy efficiency of 0.9. This resulted from the fast increase of the inlet air temperature of the air heater, which within the first 50 s, reached 45${}^{C}$. The regenerative heat exchanger was characterized by constant exergy efficiency of 0.7. The exergy destruction in this component increased with increasing the outlet air temperature from the desorbing bed (see Figure 1 and Figure 17). The performance of FDC was recalculated to average values, and it is presented in Figure 18. The total exergy efficiency was found to be 0.577 with a total exergy destruction of 4.163 kJ/kg of dry air. Nevertheless, exergy efficiency itself should not be treated as an indicator of the sophistication of the system or the possibility of its further efficiency increase. It is very easy to artificially increase exergy efficiency by, e.g., changing the temperature of a dead state. This is also the main reason the exergy efficiency should not be directly compared to other similar systems. The components of the FDC characterized by the lowest exergy efficiencies were the air cooler $AC_{2}$ and the direct evaporative cooler DEC. However, the exergy destructions of these components were among the smallest. The improvement of the exergy efficiency of this processes will not have a crucial impact on the efficiency of the complete system. Nevertheless, in order to minimize the exergy loss in the air cooler, the recovery of outlet water exergy (mostly temperature-related) should proceed. In the case of the evaporative cooling process, DEC can be replaced by the above-mentioned, more efficient indirect evaporative cooling. The components with the highest potential to reduce exergy destruction were the fluidized beds. They were responsible for over 30% of total exergy destruction in the system (see Figure 18b), and their exergy efficiency was about 60%.

**Figure 19:**
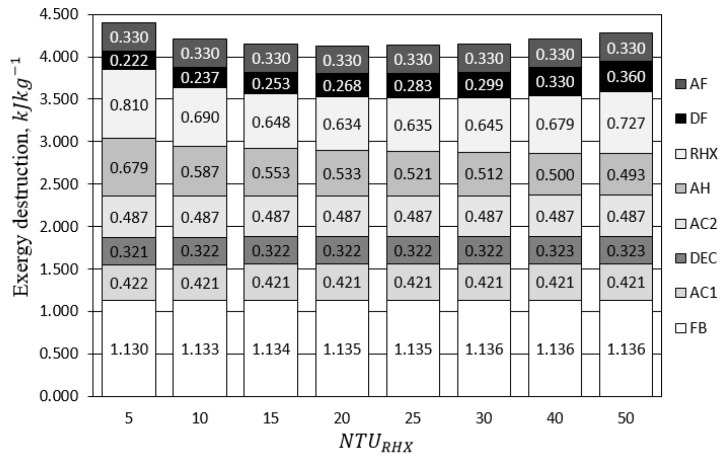
Influence of $NTU_{RHX}$ on the exergy destruction of FDC. Components abbreviations correspond to Figure 19.

**Figure 20:**
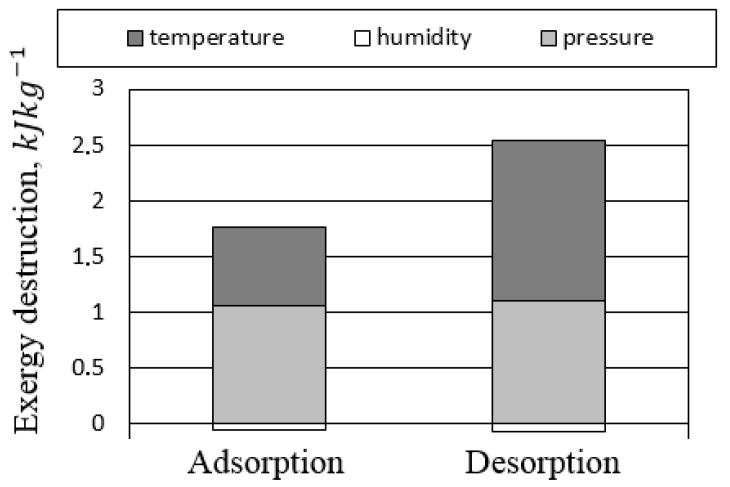
Exergy destruction related to temperature (heat transfer), pressure, and humidity.

Figure 19 presents the influence of RHX geometry $NTU_{RHX}$ on the exergy destruction of FDC components. Initially, the increase of $NTU_{RHX}$ resulted in a decrease of not only RHX, but also of AH exergy destruction. The better the RHX recovered the heat, the more efficient was the operation of AH. Therefore, the benefit of the RHX optimization was double. Increasing $NTU_{RHX}$ above 30 did not lead to a further decrease of the exergy destruction. Probably due to the increasing pressure drop in the RHX, its exergy destruction began to rise together with the total exergy destruction of the system (in spite of decreasing exergy destruction in AH). As is indicated by Equation (3), the exergy of psychrometric processes consists of three elements: exergy of pressure, temperature, and humidity. Pressure-related exergy destruction is caused by the pressure drop in the system components and piping, while temperature-related exergy destruction occurs due to the heat transfer. Figure 20 presents the exergy destruction with respect to the operation mode (adsorption/ desorption) and parameter (temperature/pressure/humidity). The share of the temperature-related exergy destruction during desorption was the highest. Significant exergy destruction occurred also due to the pressure drop. The air had low density and low heat capacity. Therefore, large air flows were involved in the processes, which in turn, lead to high pressure drops. The minimization of the pressure drop can lead to significant reduction of exergy destruction.

## 5. Conclusions

A comprehensive exergy analysis of the fluidized desiccant cooling system was carried out and described in this paper. The exergy destruction and the exergy efficiency of a particular system’s components were evaluated with respect to their geometries and operation conditions. Moreover, the exergy analysis of the complete fluidized desiccant cooling system was performed including transient exergy destruction. The influence of the system components on exergy losses was assessed together with the potential to reduce these losses. At last, temperature-, pressure-, and humidity-related exergy losses were evaluated. Based on the results of the performed analyzes, the following conclusions can be drawn:

1. The total exergy destruction in FDC was 4.163 ${\mathrm{kJkg}}^{-1}$, and the exergy efficiency was found to be 0.577.
2. The main sources of exergy losses in FDC were the fluidized beds and the regenerative heat exchanger. These components were responsible for 30% and 20% of the total exergy destruction, respectively. Nevertheless, both components were characterized by relatively high exergy efficiency of 0.58 and 0.77, respectively.
3. Optimization of the RHX exergy destruction led also to the decrease of exergy losses in the air heater. For the analyzed case, the optimum $NTU_{RHX}$ was found to be about 20.
4. The most inefficient components of FDC were the direct evaporative cooler and the air cooler $AC_{2}$ (featuring an exergy efficiency of 10% and 20%, respectively).
5. In order to improve the exergy efficiency, the direct evaporative cooler can be replaced with indirect or indirect/direct evaporative cooling. However, this can result in a significant decrease of the system’s capacity.
6. The air cooler efficiency was low due to the dissipation of outlet water exergy. Recovery of this exergy flow would reduce the exergy destruction by 25%.
7. Nearly 30% of exergy destruction occurred due to the pressure drop in FDC components. This was caused by a low density and heat capacity of the air. The decrease of the overall pressure drop can significantly improve the exergy efficiency.

## Figures and Tables

**Table 2:** Parameters used during fluidized bed modeling [1,14].

Name	Symbol	Unit	Quantity
Desiccant filling height	$h_{sor}$	m	0.03
Switching time	$t_{sw}$	s	450
Superficial air velocity	$U_{des/ads}$	${\mathrm{ms}}^{-1}$	3
Desiccant particle diameter	$d_{sor}$	m	0.001
Fluidised bed height	$h_{bed}$	m	0.55
Fluidised bed diameter	$D_{bed}$	m	0.28
Desiccant density	$\rho_{sor}$	${\mathrm{kgm}}^{-3}$	850
Inlet temperature of heating water	$T_{H}$	${}^{\circ }C$	70
Inlet temperature of cooling water	$T_{M}$	${}^{\circ }C$	25

## Abbreviations

The following abbreviations are used in this manuscript:

Acronyms		
COP	Coefficient of performance	
AC	Air cooler	
AH	Air heater	
AF	Adsorption circuit fan	
DF	Desorption circuit fan	
RHX	Regenerative heat exchanger	
DEC	Direct evaporative cooler	
Greek symbols		
η	Efficiency	
ν	Dynamic viscosity	${\mathrm{Pas}}^{-1}$
ρ	Density	${\mathrm{kgm}}^{-3}$
Symbols		
*A*	Heat exchange surface	$m^{2}$
*c*	Specific heat	${\mathrm{Jkg}}^{-1}K^{-1}$
*d*	Channel dimension of the RHX	m
ex	Specific exergy	${\mathrm{Jkg}}^{-1}$
${\dot{ex}}_{dest}$	Specific exergy destruction	${\mathrm{Jkg}}^{-1}$
*E*	Parameter of the heat exchanger	-
$\dot{Ex}$	Flow of exergy	W
${\dot{Ex}}_{dest}$	Exergy destruction	W
*F*	Correction factor	-
*h*	Specific enthalpy	${\mathrm{Jkg}}^{-1}$
$H_{ev}$	Heat of evaporation	${\mathrm{Jkg}}^{-1}$
*k*	Overall heat transfer coefficient	${\mathrm{Wm}}^{-2}K^{-1}$
*l*	Channel dimension of the RHX	m
$\dot{m}$	Mass flow	${\mathrm{kgs}}^{-1}$
NTU	Number of heat transfer units	-
*p*	pressure	Pa
*P*	Temperature ratio	-
*Q*	Heat flow	W
*R*	Individual gas constant	${\mathrm{Jmol}}^{-1}K^{-1}$
$R_{c}$	Heat capacity ratio	-
*s*	Specific entropy	${\mathrm{Jkg}}^{-1}K^{-1}$
*T*	Temperature	K
*U*	Velocity	${\mathrm{ms}}^{-1}$
*w*	Channel dimension of the air heater/cooler	m
*X*	Specific humidity ratio	${\mathrm{kgkg}}^{-1}$
$\bar{X}$	Specific humidity ratio on a molar basis	${\mathrm{molmol}}^{-1}$
Subscripts		
0	Dead state	
*a*	Air	
Carnot	The Carnot cycle	
dest	Destruction	
ex	Exergy	
*H*	Heat source	
in	Inlet	
*L*	Cooling effect	
*M*	Heat sink	
*v*	Vapor	
*w*	Water	
